# The deubiquitinating enzyme UCHL1 promotes resistance to pemetrexed in non-small cell lung cancer by upregulating thymidylate synthase

**DOI:** 10.7150/thno.42096

**Published:** 2020-05-15

**Authors:** Xinyuan Ding, Yuting Gu, Min Jin, Xin Guo, Sudong Xue, Caihong Tan, Jiefang Huang, Wanlin Yang, Mingxing Xue, Qianjun Zhou, Wenjuan Wang, Yanyun Zhang

**Affiliations:** 1 Children's Hospital of Soochow University, Institutes for Translational Medicine, State Key Laboratory of Radiation Medicine and Protection, Medical College of Soochow University, Soochow University, Suzhou 215000, China.; 2 Department of Pharmacy, the Affiliated Suzhou Hospital of Nanjing Medical University, Suzhou 215000, China.; 3 CAS Key Laboratory of Tissue Microenvironment and Tumor, Shanghai Institute of Nutrition and Health, Shanghai Institutes for Biological Sciences, University of Chinese Academy of Sciences, Chinese Academy of Sciences, Shanghai 200127, China.; 4 Department of Pharmacy, The Affiliated Hospital of Jiangsu University, Zhenjiang 212000, China.; 5 Shanghai Lung Cancer Center, Shanghai Chest Hospital, Shanghai Jiao Tong University School of Medicine, Shanghai 200030, China.

**Keywords:** non-small cell lung cancer, chemoresistance, pemetrexed, UCHL1, thymidylate synthase

## Abstract

**Rationale**: Resistance to pemetrexed (PEM)-based chemotherapy is a major cause of progression in non-small cell lung cancer (NSCLC) patients. The deubiquitinating enzyme UCHL1 was recently found to play important roles in chemoresistance and tumor progression. However, the potential roles and mechanisms of UCHL1 in PEM resistance remain unclear.

**Methods**: Bioinformatics analyses and immunohistochemistry were used to evaluate UCHL1 expression in NSCLC specimens. Kaplan-Meier analysis with the log-rank test was used for survival analyses. We established PEM-resistant NSCLC cell lines by exposing them to step-wise increases in PEM concentrations, and *in vitro* and *in vivo* assays were used to explore the roles and mechanisms of UCHL1 in PEM resistance using the NSCLC cells.

**Results**: In chemoresistant tumors from NSCLC patients, UCHL1 was highly expressed and elevated UCHL1 expression was strongly associated with poor outcomes. Furthermore, UCHL1 expression was significantly upregulated in PEM-resistant NSCLC cells, while genetic silencing or inhibiting UCHL1 suppressed resistance to PEM and other drugs in NSCLC cells. Mechanistically, UCHL1 promoted PEM resistance in NSCLC by upregulating the expression of thymidylate synthase (TS), based on reduced TS expression after UCHL1 inhibition and re-emergence of PEM resistance upon TS restoration. Furthermore, UCHL1 upregulated TS expression, which mitigated PEM-induced DNA damage and cell cycle arrest in NSCLC cells, and also conferred resistance to PEM and other drugs.

**Conclusions**: It appears that UCHL1 promotes PEM resistance by upregulating TS in NSCLC cells, which mitigated DNA damage and cell cycle arrest. Thus, UCHL1 may be a therapeutic target for overcoming PEM resistance in NSCLC patients.

## Introduction

Non-small cell lung cancer (NSCLC) predominantly involves adenocarcinoma and accounts for approximately 85% of lung cancers 1. Pemetrexed (PEM) is an antifolate that is widely used for first-line chemotherapy in clinical practice 2. However, both intrinsic and acquired resistance to PEM frequently occur, which is associated with a poor prognosis among NSCLC patients 3. Therefore, it is important to better understand the mechanisms underlying PEM resistance, which may help improve its clinical use.

The PEM molecule inhibits the activity of folate-dependent enzyme thymidylate synthase (TS), which is required for de novo synthesis of nucleotides used during DNA replication 4. Therefore, PEM blocks DNA replication and leads to cell cycle arrest, which inhibits tumor growth 5. However, elevated TS levels in NSCLC cells may render them insensitive to PEM 6, which may be related to the higher TS levels reducing the activity of PEM and weakening its efficacy. In addition, PEM can kill tumors by inducing DNA damage 7, whereas a tumor's chemotherapy resistance is often achieved through increased DNA repair 8. Thus, there is an urgent need for a better understanding of the mechanisms that regulate PEM resistance, which may help identify new therapeutic targets.

Ubiquitination and deubiquitination are reversible post-translational modifications that rely on ubiquitin ligases and deubiquitinating enzymes (DUBs), and are involved in most areas of cell biology 9. The role of deubiquitination in chemoresistance has been explored in numerous studies 10, 11, and we have previously demonstrated that ubiquitin C-terminal hydrolase L1 (UCHL1), which is a type of DUB, was associated with a favorable prognosis in neuroblastoma 12. We have also reported that UCHL1 was critical for the survival and immunosuppressive function of mesenchymal stromal cells 13. Other recent studies have found that UCHL1 could promote tumor cell proliferation and inhibit cell cycle arrest 14, which are involved in the development of chemoresistance in cervical cancer and pancreatic cancer 15, 16. However, it remains unclear whether UCHL1 is involved in regulating PEM resistance in NSCLC. Therefore, we aim to identify that the UCHL1 expression profiles in NSCLC specimens, as well as in two parental and PEM-resistant NSCLC lines. Genetic silencing or inhibition of UCHL1 was also tested to explore the roles and mechanisms of UCHL1 in PEM resistance using PEM-resistant NSCLC cells. Our findings indicate that UCHL1 plays a vital role in the development of PEM resistance in NSCLC cells, and that this protein may be a useful target for pharmacological strategies that aim to overcome PEM resistance.

## Materials and Methods

### Patients and NSCLC specimens

The present study evaluated a tissue microarray (TMA) that was performed from surgical specimen after neoadjuvant chemotherapy in 63 patients with primary NSCLC. The patients had firstly biopsy-confirmed stage Ⅲa-Ⅲb disease, had not been treated using chemotherapeutic or molecularly targeted agents, and then underwent preoperative neoadjuvant chemotherapy based on the NSCLC guidelines at the Affiliated Hospital of Jiangsu University (Zhenjiang, China) between March 2012 and February 2014. According to the Response Evaluation Criteria in Solid Tumors (RECIST, version 1.1), the NSCLC patients and their specimens were considered “chemosensitive” if they had complete or partial response or “chemoresistant” if they had progressive disease. All patients provided informed consent for the specimen collection and analysis. All experimental protocols were approved by the ethics committee of the Affiliated Hospital of Jiangsu University and complied with the principles of the Declaration of Helsinki.

### Immunohistochemistry

The immunohistochemistry (IHC) was performed using a standard immunoperoxidase staining procedure to detect UCHL1 expression in paraffin-embedded NSCLC specimens. The primary antibody was a rabbit anti-human UCHL1 antibody (1:400; Cell Signaling Technology, Danvers, Massachusetts, USA), and secondary staining was performed using an anti-rabbit secondary antibody and the DAKO ChemMate^TM^ Envision^TM^ Detection Kit (DAKO A/S, Denmark). Positive staining for UCHL1 (brown) was mainly localized in the cytoplasm. The IHC staining was scored using an H-score system based on both the staining intensity and the percentage of UCHL1-positive tumor cells. The staining intensity was scored as negative (0), weak (1+), moderate (2+), and strong (3+). The H-score was calculated using the following formula: 1 × (percentage of cells stained weakly [1+]) + 2 × (percentage of cells stained moderately [2+]) + 3 × (percentage of cells stained intensely [3+]), with overall scores ranging from 0 to 300 17. For dichotomization dividing the cohort into 2 subgroups according to chemotherapy response, the UCHL1 expressions in NSCLC patients were evaluated by the R statistical environment using the “survival ROC” package to determine the optimal cut-off value for defining high or low UCHL1 expression 18.

### Validation using human databases

Detailed procedure was as described in a previous study 12. Clinical data from NSCLC patients were obtained from the R2 Genomics Analysis and Visualization Platform (R2 Platform, http://r2.amc.nl) using a publicly available TCGA database (includes 515 lung adenocarcinoma samples). After excluding patients with no information regarding treatment outcomes, we selected 220 patients with lung adenocarcinoma who underwent neoadjuvant chemotherapy (Table S1). Clinicopathological characteristics related to UCHL1 expression were analyzed via multivariate regression analysis (Table S2). Kaplan-Meier survival analysis was performed using the Kaplan Meier-plotter database, which contains data from various comprehensive sources (https://kmplot.com/analysis/, 201387_s_at, containing 720 samples).

### Establishment of pemetrexed-resistant NSCLC cells

We purchased H1299 cells and A549 cells from the American Type Culture Collection (ATCC, USA). Pemetrexed-resistant (PEM-R) cell lines were named H1299/PEM and A549/PEM, and were established by exposing the parental cell lines to step-wise increases of PEM (MedChemExpress, Monmouth Junction, NJ, USA) at the 50% inhibitory concentration (IC50) over a 6-month period 19, 20. The PEM-R NSCLC cells were confirmed to have acquired stable resistance and were used for the subsequent experiments.

### Cell proliferation

Cell proliferation was monitored using the Cell Counting Kit-8 (CCK-8; Dojindo, Kumamoto, Japan), according to the manufacturer's instructions.

### Real-time quantitative PCR

Detailed procedures for these steps have been previously reported 12. The primer sequences used for the PCR analysis were listed in Table S3. All primers were synthesized by Sangon Biotech (Shanghai, China).

### Western blot analysis

Detailed procedure was as described in a previous study 21. Primary anti-human antibodies against UCHL1, TS, ERCC1, c-Myc, Caspase 3, Ubiquitin, Cyclin D1, and GAPDH were all purchased from Cell Signaling Technology.

### Immunofluorescence

Cells were grown on glass coverslips and then fixed with 4% paraformaldehyde. After a PBS wash, the cells were permeabilized using 0.1% Triton X-100, incubated in a blocking solution (PBS with 3% bovine serum albumin), and further incubated overnight at 4 °C with rhodamine phalloidin (Cytoskeleton, Denver, Colorado, USA) and the primary antibody to UCHL1 (Cell Signaling Technology). The fluorescent conjugated secondary antibody was Alexa Fluor 488 (Invitrogen, Carlsbad, California, USA), and DAPI (Sigma Aldrich, St Louis, MO) was used as a nuclear counterstain for 10 min. The coverslips were finally mounted onto slides with fluorescent mounting medium and immediately observed via confocal microscopy.

### Lentiviral vector construction

Silencing of gene expression was achieved using short hairpin RNA (shRNA) technology. The shRNAs targeting human *UCHL1* (sh*UCHL1*; 5'-GATCCCGGGTAGATGACAAGGTGAATCTCGAGATTCACCTTGTCATCTACCCGTTTTTG-3'; scrambled control (shNC): 5'-GATCCCCTAAGGTTAAGTCGCCCTCGCTCGAGCGAGGGCGACTTAACCTTAGGTTTTTG-3'; Sangon Biotech) were cloned into the PLVX-GFP plasmid, which was a gift from Bob Weinberg (Addgene, Cambridge, USA). High-titer lentiviral stocks were produced in HEK293T cells using Lipofectamine 2000 (Life Technologies, Darmstadt, Germany) according to the manufacturer's protocol, and the A549/PEM and H1299/PEM cells were infected with the shNC and sh*UCHL1* lentiviruses using polybrene (Life Technologies) according to the manufacturer's protocol.

Full-length *TS* cDNAs were synthesized by Genscript (Nanjing, China). These cDNAs were subcloned into pLVX-IRES-ZsGreen1 vectors (YouBio, Shanghai, China) containing an N-terminal His epitope tag. The NSCLC cells were transfected with an empty vector lentivirus (VEC) or the *TS*-containing lentivirus (*TS*) using polybrene.

### Flow cytometry

The flow cytometry (FCM) was performed using a cell cycle analysis kit (Dojindo) according to the manufacturer's protocol and analyzed by a FACS Calibur flow cytometer (BD Biosciences, San Jose, CA, USA).

### TS enzyme activity assay

The TS enzyme activity analysis was conducted using a human TS ELISA Kit (Lanpai, Shanghai, China) according to the manufacturer's protocol. The TS concentration and total TS enzyme activity were analyzed using the culture supernatant and a microtiter plate reader within 15 min. The TS enzyme activity per pmol (×10^3^ U/pmol) was calculated by dividing the total TS enzyme activity (U/mL) by the concentration of TS (pmol/L).

### Animal experiments

The protocols for all animal experiments were approved by the committee for animal experimentation of Soochow University. The H1299 and H1299/PEM cells (5 × 10^6^ cells) were injected subcutaneously into the right flanks of 5-week-old BALB/c nu/nu mice that were purchased from SLAC (Shanghai, China). These mice were monitored every other day until the tumor volume reached 100 mm^3^, and then the mice were randomly divided into 6 groups to receive the different treatments. The mice received weekly intraperitoneal treatments involving 100 mg/kg PEM 22, 0.4 mg/kg of a UCHL1 inhibitor (LDN-57444; Selleckchem, Houston, USA), or the vehicle (10% DMSO in PBS) 23. The H1299/PEM-shNC and H1299/PEM-sh*UCHL1* cells were followed with above steps without administration with LDN-57444. Finally, the mice were sacrificed for subsequent experiments when they reached the end.

### Statistical analysis

The statistical analyses were performed using IBM SPSS software (version 20) and GraphPad Prism software (version 7). All measurement data were presented as mean ± standard error. The Mann-Whitney test and analysis of variance were used to compare continuous variables. Relationships between UHCL1 expression and clinicopathological characteristics were evaluated using the χ^2^ test or Fisher's exact test. Survival curves were created using the Kaplan-Meier method and compared using the log-rank test. Differences were considered statistically significant at *p*-values of <0.05.

## Results

### High UCHL1 expression was associated with chemoresistance and poor clinical outcomes in NSCLC patients

**Figure 1:**
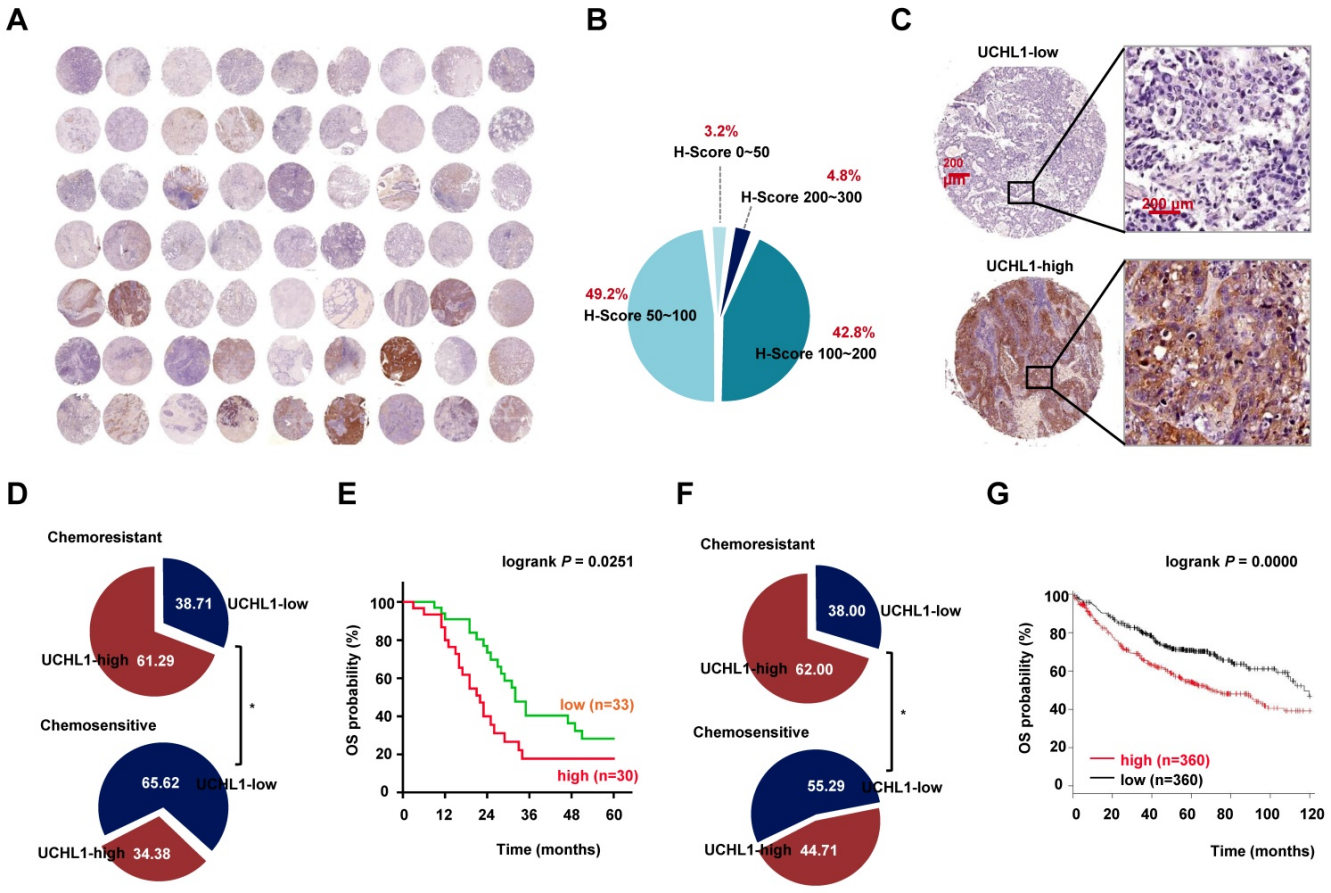
** Expression of ubiquitin C-terminal hydrolase L1 (UCHL1) and its clinical significance in non-small cell lung cancer (NSCLC).** (A) The expression of UCHL1 was evaluated in a tissue microarray (TMA) involving 63 NSCLC samples. (B) The immunohistochemistry scores (0-300) for UCHL1 expression in the NSCLC samples were calculated and the proportions are shown. (C) Representative images showing different UCHL1 levels (40×, red bar: 200 μm). Comparison of the responses to chemotherapy according to UCHL1 expression in patients from the TMA (D) and from the TCGA database (F). Statistical analyses were performed using the χ^2^ test or Fisher's exact test. Kaplan-Meier analysis of overall survival (OS) according to UCHL1 expression among patients from the TMA (E) and from the Kaplan Meier-plotter database (G). Statistical analyses were performed using the log-rank test. *p < 0.05.

We evaluated the expression of UCHL1 based on the TMA using specimens from NSCLC patients. The results revealed that UCHL1 was differentially expressed in NSCLC patients (Figure 1A-B), with an IHC score cut-off value of 122.5 used to classify the expressions as UCHL1-high (30 patients) or UCHL1-low (33 patients, Figure 1C). In addition, the patients were categorized as chemosensitive (32 patients) or chemoresistant (31 patients) based on their responses to clinical treatment. Patients with high UCHL1 expression had a higher rate of resistance to neoadjuvant chemotherapy (Figure 1D), although UCHL1 expression was not significantly associated with any of the other clinicopathological characteristics (Table 1). Kaplan-Meier analysis revealed that patients with high UCHL1 expression from the TMA cohort had poorer overall survival (OS, Figure 1E). Based on cases from the publicly available databases, we further confirmed that high expression of UCHL1 in lung adenocarcinoma was associated with a higher rate of chemoresistance and a lower rate of OS (Figure 1F-G). The expression of UCHL1 in NSCLC independently predicted the chemotherapy response, and no significant difference in the response was observed according to the chemotherapeutic regimens (Table S2 and S4). These results suggested that UCHL1 was a prognostic marker and positively associated with chemoresistance in NSCLC.

### UCHL1 was upregulated in PEM-R NSCLC cells

**Figure 2:**
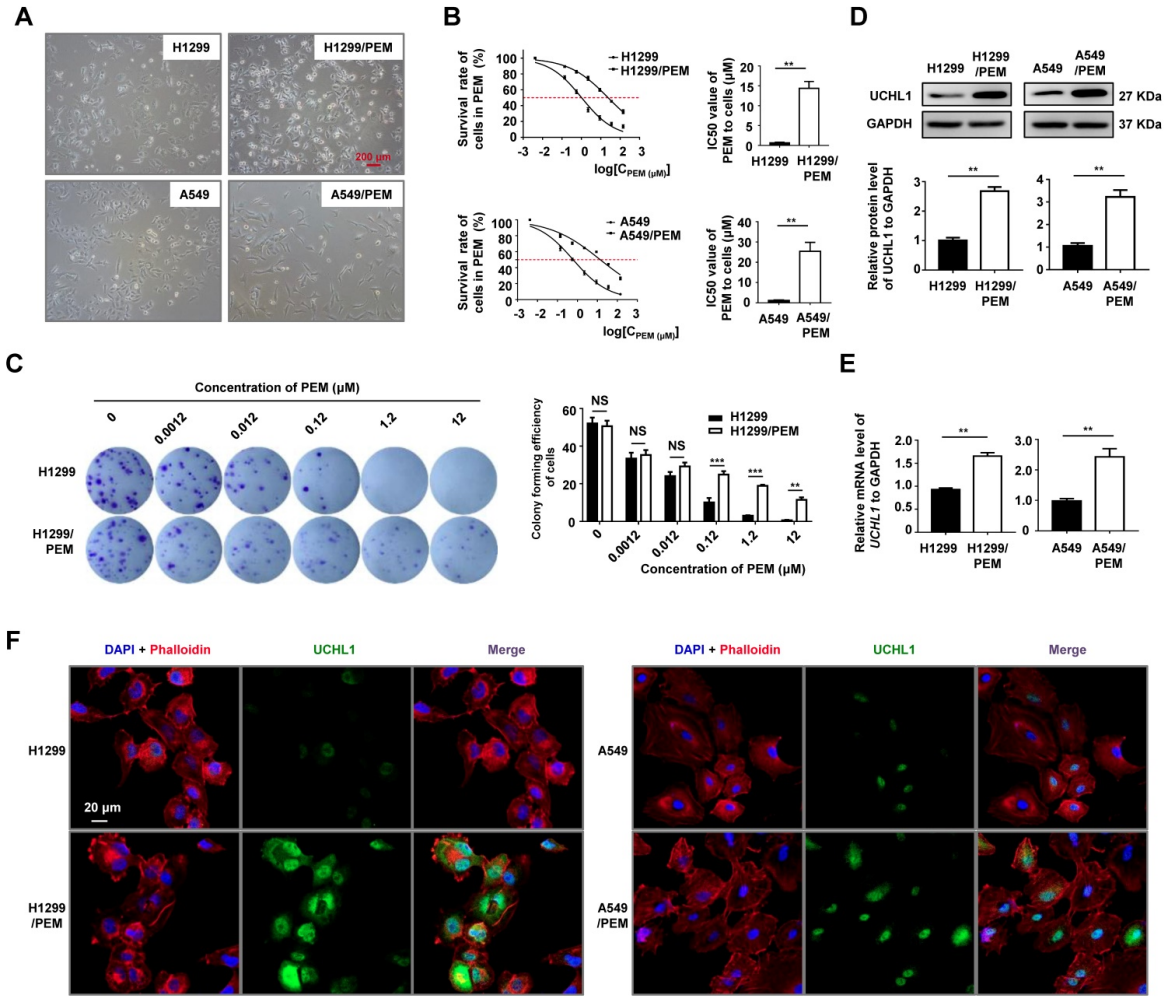
** Expression of UCHL1 in pemetrexed-resistant (PEM-R) cells.** (A) Representative micrographs of two PEM-R cell lines (10×, red bar: 200 μm). (B) Cell viability curves for the two PEM-R cell lines and their parental cell lines after PEM treatment were evaluated using the Cell Counting Kit-8 assay (left panel). The IC50 values were analyzed using the Mann-Whitney test (n = 5, right panel). (C) Colony formation assay using H1299 and H1299/PEM cells treated for two weeks using PEM or DMSO, with the results evaluated using analysis of variance (n = 5). Western blot analysis (D) and real-time quantitative PCR analysis (E) of UCHL1 levels in PEM-R cells and their parental cells, with the results analyzed using the Mann-Whitney test (n = 5). (F) Immunofluorescence assay showing the expression and intracellular location of UCHL1 in NSCLC cells (white bar: 20 μm). NS: not statistically significant, ^**^p < 0.01, ^***^p < 0.001.

We established two PEM-R cell lines (H1299/PEM and A549/PEM, Figure 2A), and these cells were more elongated and had more projections than the parental cells, without any significant changes in cell sizes. Relative to the parental cells, the H1299/PEM and A549/PEM cells had significantly increased IC50 values (Figure 2B), with resistant indexes of 23.99 ± 3.80 for the H1299/PEM cells and 23.51 ± 2.90 for the A549/PEM cells. Colony formation assays also indicated that the H1299/PEM and A549/PEM cells exhibited higher proliferation rates than their parental cells in the presence of PEM (Figure 2C and S1A). The growth rates of the PEM-R cells were comparable to those of the parental cells, with the PEM resistance persisting for a considerable period of time (Figure S1B-C). As expected, the mRNA and protein levels of UCHL1 in the PEM-R cells were significantly increased, relative to in the parental cells (Figure 2D-E). In addition, immunofluorescence staining confirmed that increased UCHL1 levels were observed in both the cytoplasm and the nucleus of the PEM-R cells (Figure 2F and S2). Thus, UCHL1 expression was upregulated in the PEM-R NSCLC cells.

### UCHL1 conferred resistance to PEM and other drugs in NSCLC cells

**Figure 3:**
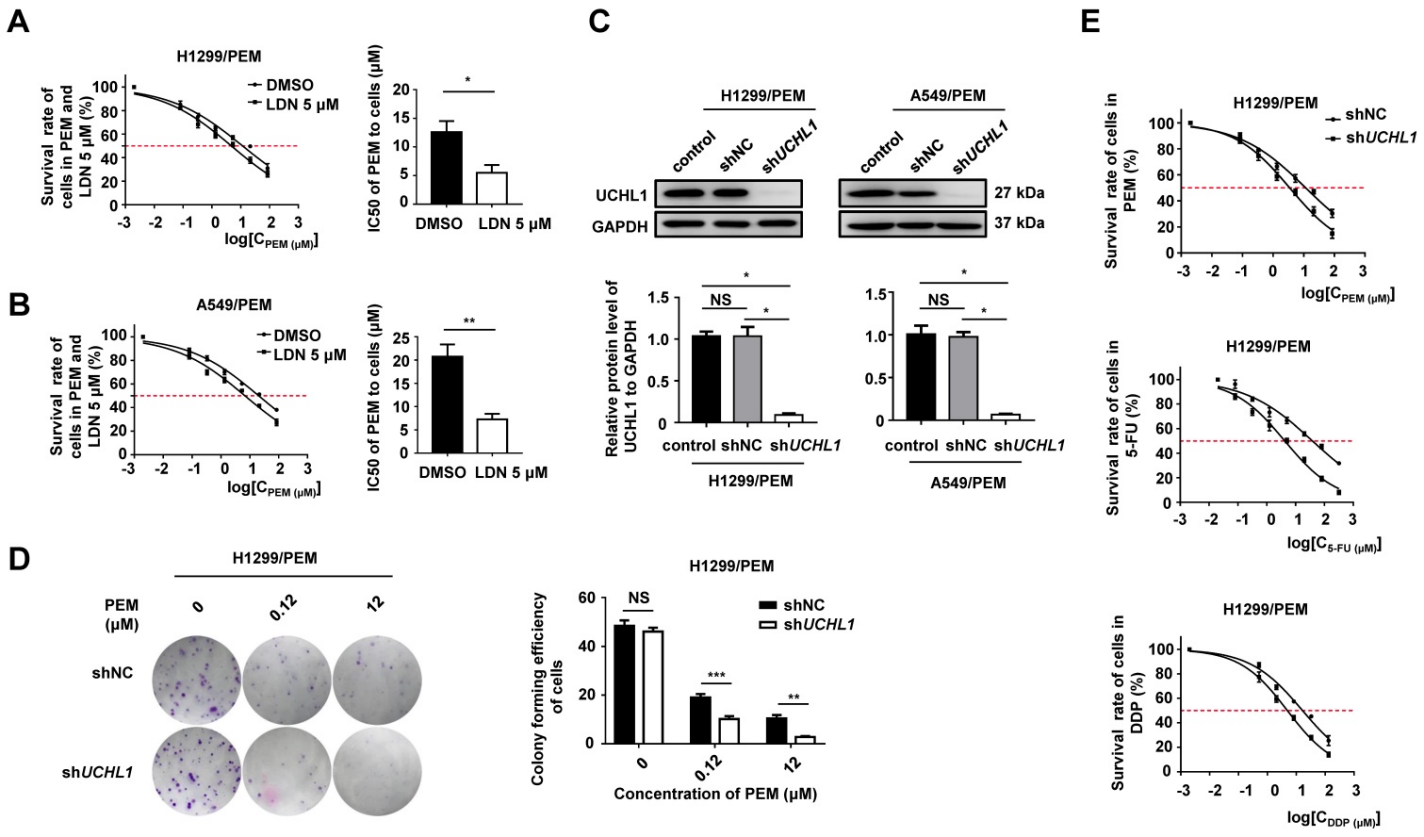
** Effect of UCHL1 expression on chemoresistance in NSCLC cells.** The IC50 values for PEM-treated H1299/PEM cells (A) and A549/PEM cells (B) after exposure to 5 μM LDN (a UCHL1 inhibitor) or DMSO (n = 5). (C) Western blot showing UCHL1 expression in PEM-R cells after UCHL1 silencing (n = 5). (D) Colony formation assay showing the proliferation rate of H1299/PEM cells treated using PEM or DMSO for 2 weeks (left panel), with the results evaluated using analysis of variance (n = 5, right panel). (E) Cell viability curves for H1299/PEM-shNC and H1299/PEM-sh*UCHL1* cells treated using PEM, 5-FU, or DDP. The statistical results are shown in Table 2. NS: not statistically significant, ^*^p < 0.05, ^**^p < 0.01, ^***^p < 0.001.

We used a selective inhibitor of UCHL1 (LDN-57444, referred to as LDN hereafter) 23 to treat the PEM-R NSCLC cells, and found that LDN promoted protein ubiquitination but had almost no effect on cell proliferation when it was administered alone (Figure S3A-C). However, the IC50 values for the two PEM-R cell lines sharply decreased when PEM was administered with LDN (Figure 3A-B). Furthermore, we found that UCHL1 silencing in PEM-R cells dramatically decreased cell clonality (Figure 3C-D and S3D) and increased chemosensitivity (Figure 3E). Based on these findings, it appears that UCHL1 was critical for maintaining PEM resistance in NSCLC cells.

It is possible that if tumor cells are resistant to one drug, they will also be resistant to many other drugs 24. We observed that, relative to the parental cells, the PEM-R cells had significantly higher IC50 values for most first-line and second-line drugs that we tested (Table S5). Interestingly, the resistance indexes for both PEM-R cell lines were >10.0 for both 5-FU and cisplatin (DDP). In addition, UCHL1 silencing in the PEM-R cells dramatically decreased the IC50 values for 5-FU and DDP (Table 2). Therefore, UCHL1 appears to play important roles in multidrug resistance.

### UCHL1 facilitated PEM resistance by maintaining cell cycle progression and enhancing DNA repair

**Figure 4:**
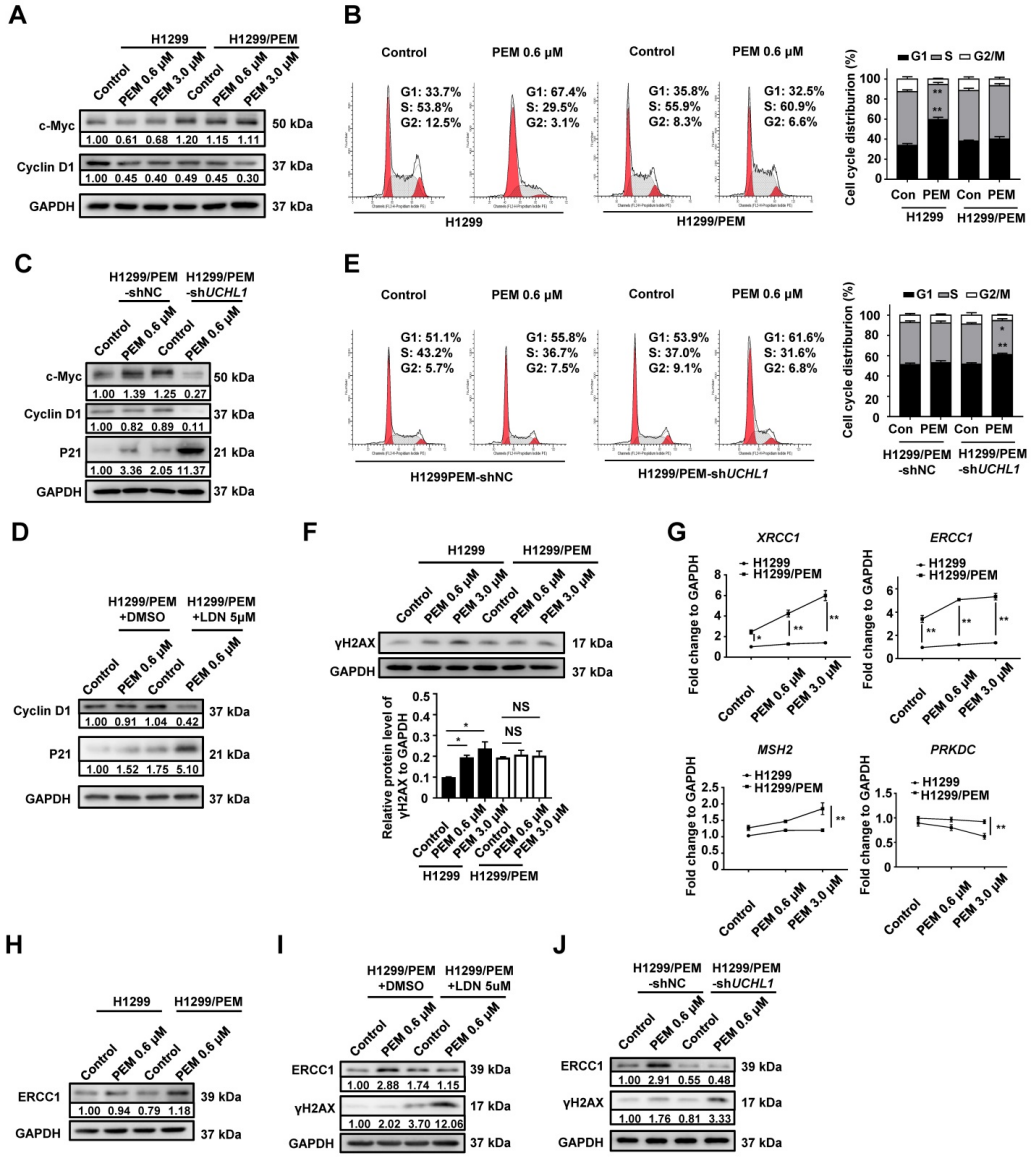
** Expression of UCHL1 maintained cell cycle progression and enhanced DNA repair.** In the presence of PEM or DMSO, western blot was used to evaluate the levels of cell cycle-associated proteins in H1299 cells and H1299/PEM cells (A), and H1299/PEM cells with UCHL1 silencing (C) or inhibition (D). Flow cytometry results showing the cell cycle changes in H1299 cells and H1299/PEM cells (B), and H1299/PEM cells with UCHL1 silencing (E). (F) Western blot showing γH2AX levels in NSCLC cells treated using PEM or DMSO (n = 5). (G) The mRNA levels of DNA repair enzymes in NSCLC cells were evaluated using real-time quantitative PCR (n = 5, *PRKDC:* protein kinase, DNA activated, catalytic polypeptide, *MSH2:* MutS homolog 2). Western blot showing the levels of ERCC1 and γH2AX in H1299 cells and H1299/PEM cells (H), and H1299/PEM cells with UCHL1 inhibition (I) or silencing (J). ^*^p < 0.05, ^**^p < 0.01.

As PEM is an antifolate drug that can induce cell cycle arrest in NSCLC cells 25, we evaluated the role UCHL1 in regulating the cell cycle after PEM treatment. Interestingly, we found that c-Myc and Cyclin D1 (cell cycle-related proteins) had lower expression in the parental cells than in the PEM-R cells (Figure 4A and S4A). Furthermore, FCM revealed that PEM treatment of the parental cells was associated with a significantly increased proportion of cells in the G1 phase and a decreased proportion of cells in the S phase. However, PEM treatment did not affect the cell cycle in the PEM-R cells (Figure 4B and S4B). In contrast, UCHL1 silencing or inhibition in PEM-R cells decreased the levels of c-Myc and Cyclin D1 (Figure 4C-D and S4C) and increased the levels of p21 (a cell cycle arrest-related protein). In addition, the PEM-induced G1 arrest was aggravated in UCHL1-depleted PEM-R cells (Figure 4E and S4D). These results indicated that UCHL1 was critical for sustaining cell cycle progression in the presence of PEM treatment.

A previous study has demonstrated that DUBs play crucial roles in the repair of DNA damage 26. We found that PEM treatment significantly increased the levels of γH2AX (a marker of DNA double-strand breaks) in parental cells, but that it had no effect on γH2AX levels in PEM-R cells (Figure 4F and S5A). Real-time quantitative PCR analysis of representative DNA repair-associated genes in PEM-R cells revealed significantly upregulated expression of X-ray repair cross complementing 1 (*XRCC1*, functions in base excision repair) and excision repair cross-complementing 1 (*ERCC1*, functions in nucleotide excision repair). In addition, these expressions were further upregulated after PEM treatment (Figure 4G and S5B). Consistent with the fact that DDP resistance mainly involves nucleotide excision repair 27, we found that ERCC1 expression was elevated in the two PEM-R cell lines, while UCHL1 silencing or inhibition suppressed ERCC1 expression in the PEM-R cells (Figure 4H-J and S5C-D). These results indicated that UCHL1 was required for the enhanced DNA repair that was induced in response to treatment using PEM or other drugs.

### UCHL1 promoted cell cycle progression and DNA repair through regulating TS

**Figure 5:**
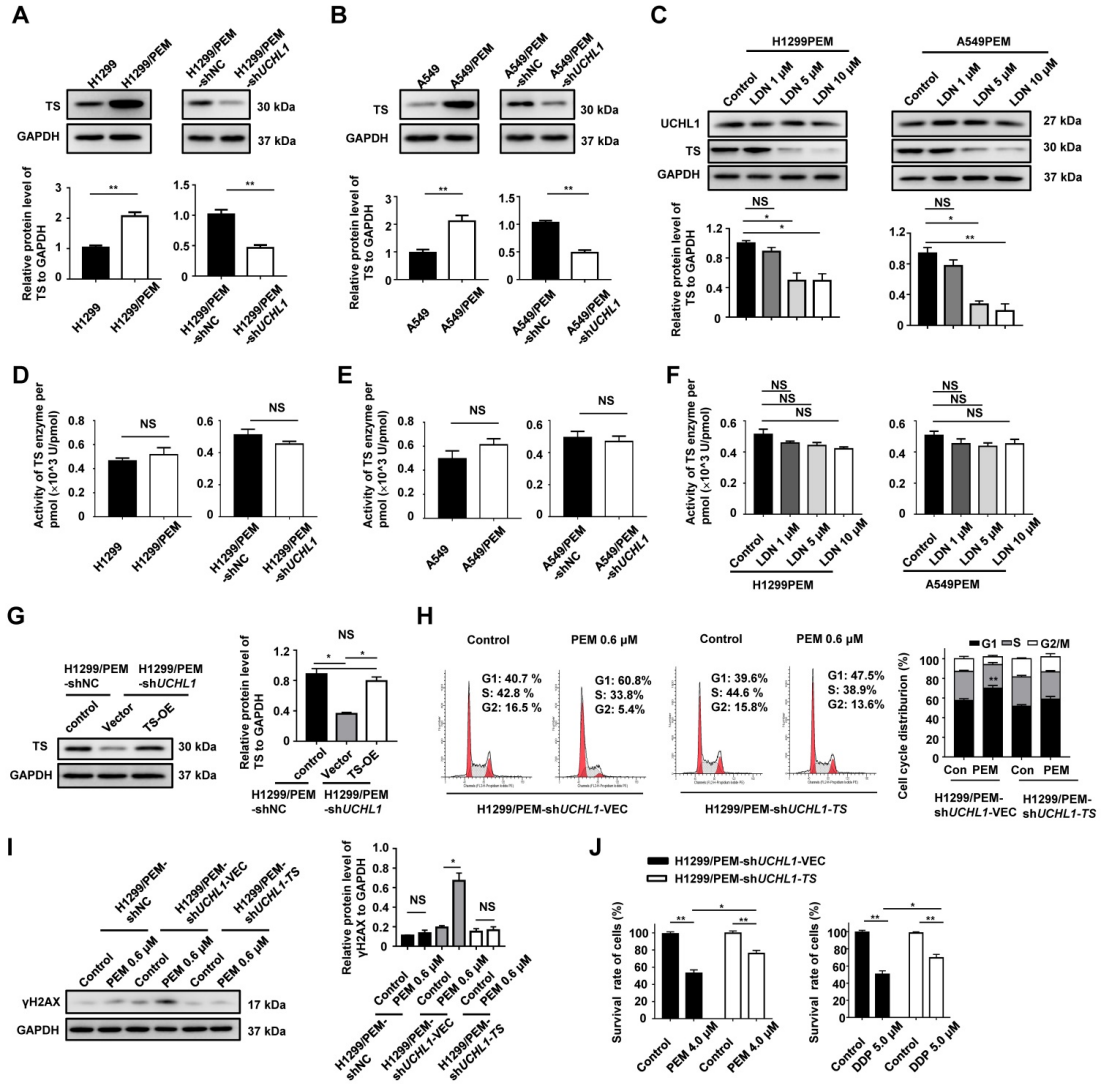
** Expression of UCHL1 promoted drug resistance through regulating thymidylate synthase (TS).** Western blot analysis of TS levels (A, B) and TS activity assay (D, E) in H1299 and its derived cells as well as in A549 and its derived cells. The TS protein level (C) and enzyme activity (F) were analyzed in PEM-R cells treated using LDN or DMSO (n = 5). Western blot showing the levels of TS (G) and γH2AX (I) in H1299/PEM-sh*UCHL1* cells transfected using either an empty vector lentivirus (-VEC) or *TS*-containing lentivirus (-*TS*). Cell cycle analysis (H) and cell viability analysis (J) were performed in the presence of PEM or DDP using H1299/PEM-sh*UCHL1*-*TS* cells (n = 5). NS: not statistically significant, ^*^p < 0.05, ^**^p < 0.01.

The TS protein catalyzes the reductive methylation of 2'-deoxyuridine-5'-monophosphate, which forms deoxythymidine monophosphate that is used in the maintenance of DNA replication and repair 28. Interestingly, we found that the TS mRNA and protein levels were remarkably upregulated in the two PEM-R cell lines, and were significantly reduced by UCHL1 silencing or inhibition (Figure 5A-C and S6A-B). In addition, no significant difference was observed in TS enzyme activity per pmol (Figure 5D-F). When TS was re-introduced into *UCHL1*-knockdown H1299/PEM cells without changing TS enzyme activity (per pmol) via transfecting TS lentivirus (designated as H1299/PEM-sh*UCHL1*-*TS*), the cell cycle progression was significantly restored (Figure 5G-H and S6C). Furthermore, no change in γH2AX levels was observed, even after PEM treatment (Figure 5I).

The survival rate of the H1299/PEM-sh*UCHL1*-*TS* cells was also significantly higher than that of the control cells in the presence of PEM or DDP (Figure 5J). These results indicated that the UCHL1/TS axis protected NSCLC cells from PEM treatment by promoting cell cycle progression and DNA repair.

### UCHL1 inhibition suppressed the growth of PEM-R xenografts

**Figure 6:**
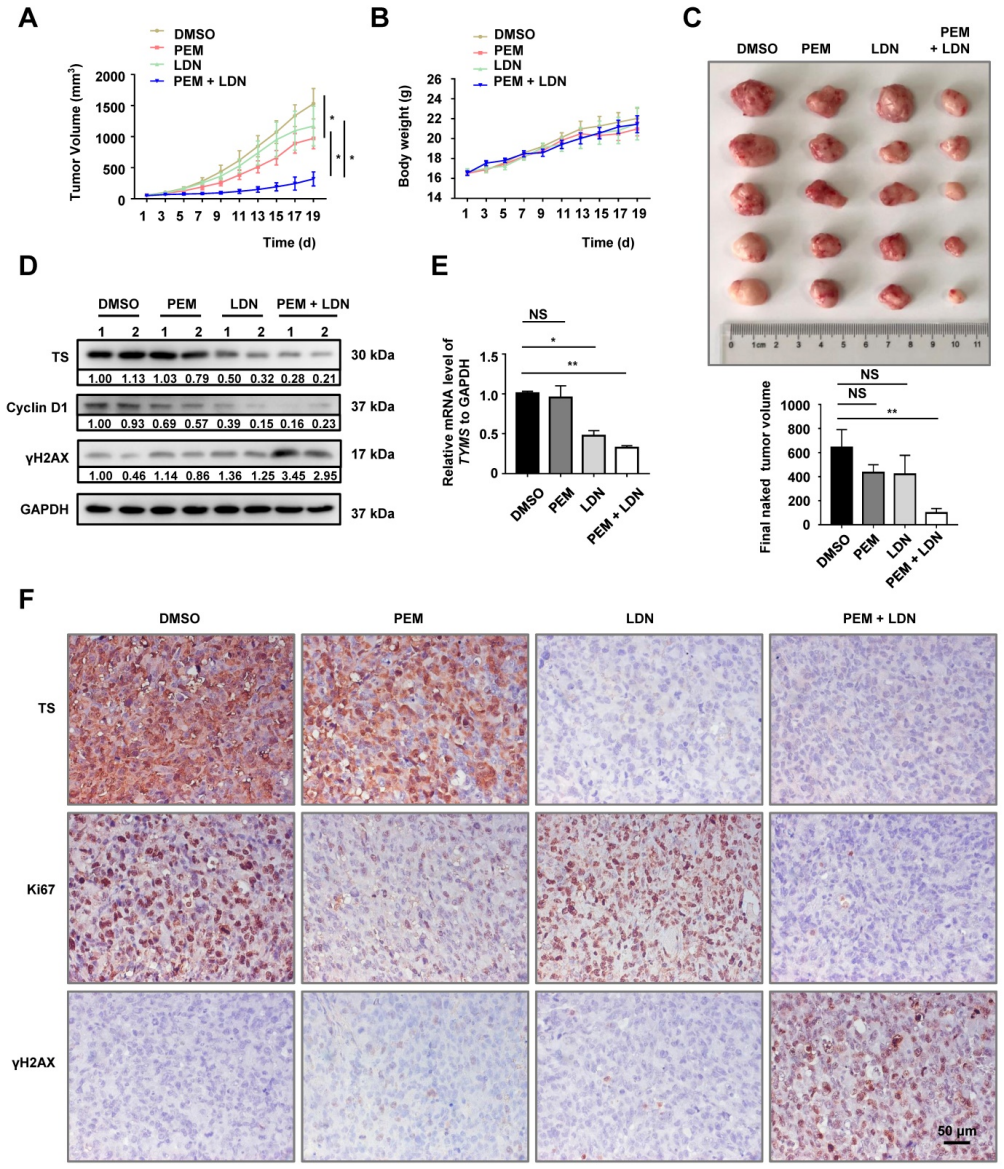
** Antitumor effects of UCHL1 antagonists in PEM-R tumor xenografts.** H1299/PEM cells were subcutaneously injected into BALB/c nu/nu mice, while followed by weekly intraperitoneally treatments using PEM, LDN, or the vehicle. The tumor sizes (A) and body weights (B) were evaluated using analysis of variance (n = 5). (C) The mice were sacrificed and the tumors were removed (upper panel) to evaluate tumor volumes using analysis of variance (bottom panel). (D) Tumor lysates were resolved and western blot was used to evaluate the levels of TS, Cyclin D1, and γH2AX. (E) The mRNA level of TS (*TYMS*) was determined using real-time quantitative PCR (n = 5). (F) Immunohistochemistry was performed to detect the levels of TS and other proteins, and photographs were obtained using a 40× objective lens (black bar: 50 μm). NS: not statistically significant, ^*^p < 0.05, ^**^p < 0.01.

We generated H1299-derived and H1299/PEM-derived xenografts in nude mice to evaluate their PEM resistance *in vivo*. Relative to the H1299-derived control group, the H1299/PEM-derived group exhibited a decreased response to PEM (Figure S7A), without a significant effect on the body weight of the mice (Figure S7B). In addition, the UCHL1 mRNA and protein levels in H1299/PEM-derived tumors were significantly higher than those in the H1299-derived tumors (Figure S7C-D). Furthermore, the combination of PEM plus LDN had a synergistic effect on tumor growth inhibition, without affecting the body weight of the mice with the H1299/PEM xenografts (Figure 6A-C). Similar results were observed for xenografts that were derived from the H1299/PEM-sh*UCHL1* cells (Figure S8A-C).

The TS mRNA and protein levels were reduced by LDN treatment (Figure 6D-E). Furthermore, in contrast with the findings from treatment using PEM or LDN alone, the combination of PEM plus LDN reduced the levels of Cyclin D1 and elevated the levels of γH2AX. The IHC findings also indicated that TS levels were reduced after LDN treatment. Moreover, the combination of LDN plus PEM inhibited cell proliferation more effectively, based on Ki67 staining, and induced more DNA damage (Figure 6F). The findings indicated that UCHL1 was essential for the PEM resistance of NSCLC cells* in vivo*.

## Discussion

**Figure 7:**
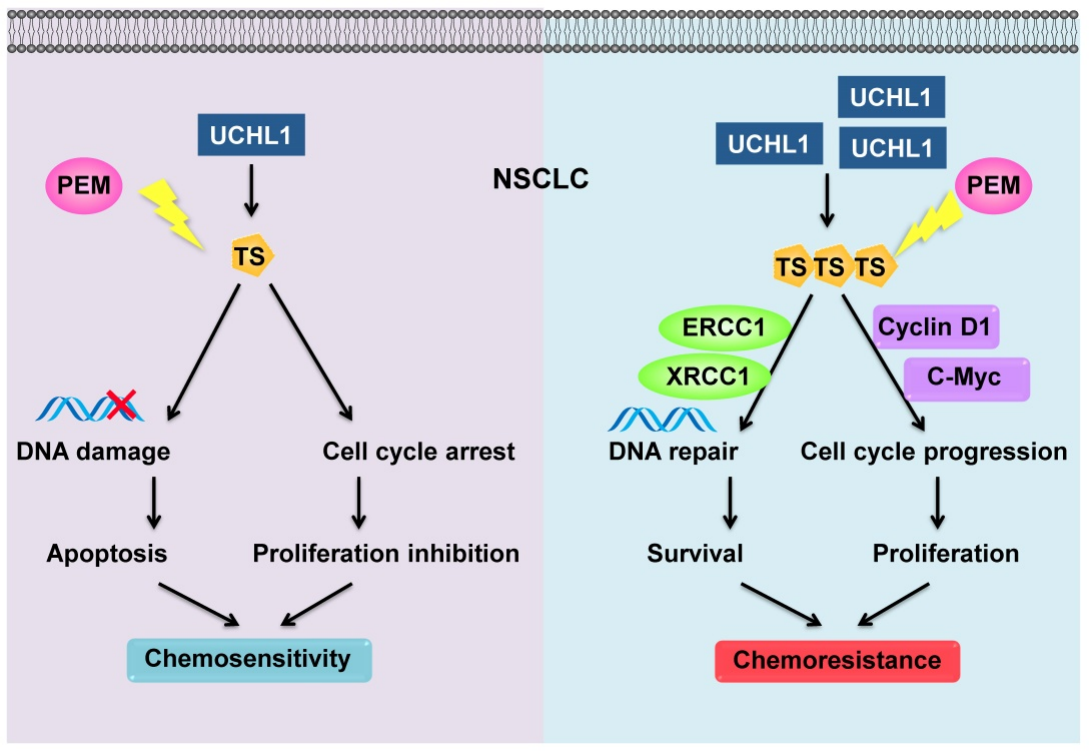
Schematic diagram of the mechanism through which UCHL1 confers PEM resistance in NSCLC.

The combination of chemotherapy, antiangiogenic agents, and immunotherapy has recently exhibited synergistic anticancer effects in NSCLC. As a first-line chemotherapy drug for non-squamous NSCLC, PEM plus cisplatin is still a standard of choice in patients without driver mutation 29. Nevertheless, chemoresistance remains an important challenge to this treatment. We found that levels of a deubiquitinating enzyme (UCHL1) were positively associated with a poor prognosis and PEM resistance in NSCLC patients. Similarly, UCHL1 was highly expressed in PEM-R NSCLC cells, while intervention of UCHL1 by genetic silencing or inhibition greatly improved the sensitivity of the PEM-R cells to PEM. Furthermore, we revealed that UCHL1 upregulated the expression of TS, which promotes cell cycle progression and DNA repair, thereby conferring resistance to PEM and other drugs (Figure 7).

Chemoresistance can develop through intrinsic or acquired mechanisms, such as reduced intracellular drug accumulation, modification of drug targets, and increased DNA repair 30, 31. Accumulating evidence has also indicated that DUBs play important roles in cancer progression, and especially in chemoresistance 32. For example, UCHL1 is a DUB that plays important roles in chemosensitivity to bortezomib, doxorubicin, and DDP in colorectal cancer, melanoma, and breast cancer 33, 34. However, it has been poorly understood whether UCHL1 plays a role in the PEM chemoresistance of NSCLC. We found that levels of UCHL1 were inversely associated with chemosensitivity, regardless of the chemotherapeutic agent, in NSCLC patients, and confirmed that UCHL1 helped mediate resistance to PEM, 5-FU, and DDP in NSCLC cells. The related mechanisms for this resistance appear to involve UCHL1 promoting cell cycle progression and DNA repair. It was indicated that UCHL1-induced chemoresistance was due to a universal mechanism. Although it was difficult to distinguish between intrinsic and acquired resistance using the human specimens, UCHL1 promoted acquired resistance in the PEM-R cells, which suggests that UCHL1 plays important roles in acquired resistance.

In patients with breast cancer, UCHL1 levels were negatively correlated with OS 35, although a separate study revealed no significant correlation in NSCLC 36. In contrast, the present study revealed that higher UCHL1 levels were associated with poor OS outcomes among patients with NSCLC. This difference may be related to the present study's larger sample size and/or the patients' clinicopathological characteristics (mainly adenocarcinoma).

TS promoted cell cycle progression and inhibited apoptosis via upregulating Cyclin E and c-Myc in lung cancer 37. Supporting this, we found that the elevated TS was dependent on UCHL1, and maintained cell cycle progression and DNA repair in PEM-R NSCLC cells. Tumor-suppressor protein p21, which was positively regulated by* p53* or negatively regulated by c-Myc, played vital roles both in the cell cycle procession 38 and senescence 39. In this study, UCHL1/TS axis increased the levels of c-Myc protein, thereby decreased p21 protein in H1299 cells with *p53* gene deletion, without a significant change of p21 protein in A549 cells with wild *p53* gene. Cellular senescence decreases the proliferation capacity of tumor cells, but is also known to enhance tumorigenesis 40. In this context, PEM treatment may induce senescence in NSCLC cells 41, however, it is possible that UCHL1 levels could be increased in senescent cells, thereby modifying the senescence process 42. In addition, PEM treatment for NSCLC may increase the levels of *TYMS* mRNA and TS protein, and induce both apoptosis 43 and autophagy bypass 44. Moreover, UCHL1 can act as an mTOR inhibitor 45, and may protect tumor cells via activation of autophagy, which could offset PEM-induced apoptosis 46. These compensatory effects of UCHL1 might also have been present in our PEM-R cells.

While the present study revealed that UCHL1 increased TS mRNA and protein expression, without any affections on TS enzyme activity (per pmol), and the underlying mechanisms remain unclear. A previous study has demonstrated that UCHL1 stabilized HIF-1α by abrogating the von Hippel-Lindau-mediated ubiquitination of HIF-1α, which subsequently promoted tumor metastasis 47. In addition, UCHL1 stabilized mTOR2 by antagonizing the DDB1-CUL4-mediated ubiquitination of raptor 48. Moreover, HIF-1α and mTOR are both closely related to TS 49, 43. Therefore, UCHL1 may indirectly promote the transcription of TS by targeting other enzymes or proteins, such as HIF-1α and mTOR, and further studies are needed to explore this topic.

In conclusion, the present study revealed that PEM resistance in NSCLC cells was depend on UCHL1. In addition, UCHL1 induced the upregulation of TS, which mitigated PEM-induced DNA damage and cell cycle arrest in NSCLC cells. Furthermore, UCHL1 conferred resistance to PEM and other drugs in these cells. Therefore, UCHL1 appears to play a critical role in the PEM resistance observed in NSCLC cells, and may be a promising therapeutic target for overcoming chemoresistance in patients with refractory NSCLC and high UCHL1 expression.

## Supplementary Material

Supplementary figures and tables.

## Figures and Tables

**Table 1:** Relationships between UCHL1 levels and clinicopathological characteristics of 63 patients with NSCLC

		N	UCHL1 expression		*p*
			Low	High	
**Total cases**		63	33	30	
**Sex**					
	Male	38	17	21	0.1342
	Female	25	16	9	
**Age (years)**					
	<60	29	12	17	0.1064
	≥60	34	21	13	
**Tobacco smoking (years × packs)**					
	≥20 (heavy)	29	13	16	0.2676
	<20 (light/never)	34	20	14	
**Histological type**					
	SCC	25	11	14	0.2800
	ADC	38	22	16	
**Differentiation**					
	Well	9	2	7	0.1048
	Moderate	47	28	19	
	Poor	7	3	4	
**Pathological TNM stage ^a^**					
	I	25	14	11	0.8459
	II	13	6	7	
	III-IV ^b^	25	13	12	
**Chemotherapy response**					
	Chemosensitive	32	21	11	0.0325^*^
	Chemoresistant	31	12	19	

N, number; SCC, squamous cell carcinoma; ADC, adenocarcinoma. Analyses were performed using the χ^2^ test or Fisher's exact test, *p < 0.05. ^a^ TNM stage of NSCLC patients here was pathological and post-operative stage. ^b^ Only one patient with ipsilateral pleural dissemination (M1a) was pathologically diagnosed with stage IVa disease.

**Table 2:** Multidrug sensitivities in the two PEM-R cell lines with UCHL1 silencing

	IC50 (μM)		*p*	IC50 (μM)		*p*
	H1299/PEM-shNC	H1299/PEM-sh*UCHL1*		A549/PEM-shNC	A549/PEM-sh*UCHL1*	
**Pemetrexed**	12.92±2.64	4.06±1.04	0.0317^*^	24.97±6.18	5.83±1.40	0.0079^**^
**5-fluorouracil**	39.84±5.96	4.54±0.93	0.0079^**^	33.37±3.46	6.89±1.58	0.0079^**^
**Cisplatin**	17.80±2.75	5.23±1.08	0.0079^**^	8.61±1.33	1.77±0.18	0.0079^**^

NC, scrambled control; IC50: 50% inhibitory concentration. Sensitivities of the NSCLC cells to the drugs were determined using the CCK-8 assay. Statistical analyses were performed using the Mann-Whitney test (n = 5), ^*^p < 0.05 or ^**^p < 0.01.

## Abbreviations

CCK-8: cell counting kit-8
DDP: cisplatin
DUBs: deubiquitinating enzymes
ERCC1: excision repair cross-complementing 1
FCM: flow cytometry
IC50: 50% inhibitory concentration
IF: immunofluorescence
IHC: immunohistochemistry
NSCLC: non-small cell lung cancer
PEM: pemetrexed
TMA: tissue microarray
TS: thymidylate synthase
UCHL1: ubiquitin C-terminal hydrolase L1
XRCC1: X-ray repair cross complementing 1
