# Supplementary material for: The deubiquitinating enzyme UCHL1 promotes resistance to pemetrexed in non-small cell lung cancer by upregulating thymidylate synthase
Source: Theranostics. 2020 May 15;10(13):6048–60. doi: 10.7150/thno.42096 (PMC7255002; doi:10.7150/thno.42096)

Supplementary Materials

Fig. S1. Cell clonality and stability analysis of the PEM-R cells. (A) Colony formation assay was performed using A549 and A549/PEM cells that were treated using PEM or DMSO as the control for 2 weeks, with the results evaluated using analysis of variance (n = 5). (B) The two PEM-R cell lines were allowed to grow or remain in culture for 8 weeks after thawing, and the resistance indexes were presented (n = 3). (C) The growth rates of PEM-R cells and their parental cells were calculated by counting the numbers of cells from day 1 to day 5, with the results evaluated using analysis of variance (n = 3). NS: not statistically significant, $^{***}P < 0.001$.

Fig. S2. The distribution of UCHL1 protein in NSCLC cells. The ratio of the intranuclear UCHL1 fluorescence intensity to the total UCHL1 fluorescence intensity in H1299 cells, H1299/PEM cells, A549 cells, and A549/PEM cells was shown and evaluated using the Mann-Whitney test (n = 3). NS: not statistically significant.

Fig. S3. Colony forming efficiency and the role of LDN57444 in the survival of NSCLC cells. (A) The ubiquitin protein levels in H1299/PEM cells after treatment for 48 h using LDN57444 (LDN) or DMSO was shown. The CCK-8 assay was used to evaluate survival of H1299 cells and H1299/PEM cells (B), and A549 cells and A549/PEM cells (C) after 48 h of treatment using LDN or DMSO, and the results were evaluated using analysis of variance (n = 3). (D) The colony formation assay was performed for A549/PEM-shNC and -sh*UCHL1* cells treated using PEM or

DMSO for 2 weeks, and the results were evaluated using analysis of variance (n = 5). NS: not statistically significant, $^{*}P < 0.05$, $^{**}P < 0.01$, $^{***}P < 0.001$.

Fig. S4. UCHL1 plays vital roles in progression of the cell cycle in A549 cells and its derived cells. In the presence of PEM or DMSO, the levels of cell cycle-associated proteins (c-Myc and Cyclin D1) were evaluated using western blot for A549 cells and A549/PEM cells (A), and A549/PEM cells with UCHL1 silencing (C). Flow cytometry was performed to evaluate changes in the cell cycle of A549 cells and A549/PEM cells (B), and A549/PEM cells with UCHL1 silencing (D), with the results evaluated using analysis of variance (n = 5). $^{*}p < 0.05$, $^{**}p < 0.01$.

Fig. S5. UCHL1 promotes DNA repair through regulating ERCC1. Western blot showing γH2AX levels (A) and ERCC1 levels (C) in NSCLC cells that were treated using PEM or DMSO for 24 h (n = 5). (B) The mRNA levels of DNA repair enzymes in NSCLC cells were determined using real-time quantitative PCR (n = 5). (D) Western blot analysis of ERCC1 and γH2AX levels in A549/PEM-shNC and A549/PEM-sh*UCHL1* cells treated using PEM and DMSO. NS: not statistically significant, $^{*}P < 0.05$, $^{**}P < 0.01$.

Fig. S6. The mRNA levels and activity of TS in NSCLC cells. Real-time quantitative PCR analysis of TS (*TYMS*) levels in H1299 and its derived cells (A) and in A549 and its derived cells (B) was shown and the results were evaluated using the Mann-Whitney test (n = 5). The enzyme activity of TS (C) was evaluated in H1299/PEM-sh*UCHL1* cells transfected using either an empty vector lentivirus (-VEC) or *TS*-containing lentivirus (-*TS*), and the results were evaluated using

analysis of variance (n = 5). NS: not statistically significant, $^{*}p < 0.05$, $^{**}p < 0.01$.

Fig. S7. H1299/PEM cells were resistant to PEM in *vivo*. The H1299 cells and H1299/PEM cells were subcutaneously injected into BALB/c nu/nu mice, which received weekly intraperitoneally treatments using 100 mg/kg PEM or the vehicle (10% DMSO in PBS). The tumor sizes (A) and body weights (B) were analyzed using analysis of variance (n = 5). (C) Tumor lysates were resolved and the UCHL1 levels were analyzed using western blot (n = 5). (D) The mRNA levels of *UCHL1* were also determined using real-time quantitative PCR (n = 5). $^{**}p < 0.01$.

Fig. S8. The roles of UCHL1 in the PEM resistance of H1299PEM cells in *vivo*. The H1299/PEM-shNC cells and -sh*UCHL1* cells were subcutaneously injected into BALB/c nu/nu mice, which received weekly intraperitoneally treatments using 100 mg/kg PEM or the vehicle (10% DMSO in PBS). The tumor sizes (A) and body weights (B) were analyzed using analysis of variance (n = 5). (C) The tumors were removed from the sacrificed mice (upper panel) and the final volumes were evaluated using analysis of variance (bottom panel). NS: not statistically significant, $^{*}p < 0.05$, $^{**}p < 0.01$.

Table S1. The relationships between UCHL1 levels and clinicopathological characteristics of 220 NSCLC patients

		N	UCHL1 expression		P
			Low	High	
**Total cases**		220	113	107	
**Sex**					
	Male	108	43	65	$P = 0.008^{**}$
	Female	112	70	42	
**Age (years)**					
	<60	71	32	39	$P = 0.1973$
	≥60	149	81	68	
**Tobacco smoking (years × packs)**					
	≥20 (heavy)	119	54	65	$^{a}P = 0.0570$
	<20 (light/never)	24	16	8	
	NA	77	43	34	
**Pathological TNM stage**					
	I–II	171	88	83	$P = 0.9565$
	III–IV [b]	49	25	24	
**Chemotherapeutics**					
	Chemosensitive	170	94	76	$P = 0.0315^{*}$
	Chemoresistant	50	19	31	

N, number; NA, not available. Analyses were performed using the χ^2 test, $^{*}p < 0.05$, $^{**}p < 0.01$.

[a] Denotes a significant difference between heavy and light/never tobacco smoking.

[b] Only three patients were pathologically diagnosed with stage Ⅳ disease.

Table S2. Multivariate analysis of clinical characteristics related to UCHL1 expression

		**OR**	**95% CI**	***P***
	**Chemotherapy response**			
	Chemosensitive	Reference		
**UCHL1 expression**	Chemoresistant	2.227	1.136–4.367	0.020*
**(high vs. low)**	**Sex**			
	Male	Reference		
	Female	0.392	0.226–0.681	0.001**

OR, odds ratio; CI, confidence interval. *$p < 0.05$, **$p < 0.01$.

Table S3. Primers used for the real-time quantitative PCR

Primer name	Sequence (5′- 3′)
human *UCHL1*	CCTGTGGCACAATCGGACTTA
	CATCTACCCGACATTGGCCTT
mouse *UCHL1*	AGGGACAGGAAGTTAGCCCTA
	AGCTTCTCCGTTTCAGACAGA
human *GAPDH*	GGAAGATGGTGATGGGATT
	GGATTTGGTCGTATTGGG
mouse *GAPDH*	AGGTCGGTGTGAACGGATTTG
	GGGGTCGTTGATGGCAACA
human *XRCC1*	TCAAGGCAGACACTTACCGAA
	TCCAACTGTAGGACCACAGAG
human *ERCC1*	CTACGCCGAATATGCCATCTC
	GTACGGGATTGCCCCTCTG
human *MSH2*	AGTCAGAGCCCTTAACCTTTTTC
	GAGAGGCTGCTTAATCCACTG
human *PRKDC*	CTGTGCAACTTCACTAAGTCCA
	CAATCTGAGGACGAATTGCCT
human *TYMS*	CTGCTGACAACCAAACGTGTG
	GCATCCCAGATTTTCACTCCCTT
mouse *TYMS*	GATTCAGATTACTCGGGACAAGG
	CAGAGCATAGCTGGCAATGT

UCHL1, ubiquitin C-terminal hydrolase L1; *GAPDH*, glyceraldehyde-3-phosphate dehydrogenase; *XRCC1*, X-ray repair cross complementing 1; *ERCC1*, excision repair cross-complementing 1; *MSH2*, mutS homolog 2; *PRKDC*, protein kinase, DNA activated, catalytic polypeptide; *TYMS*, thymidylate synthetase.

Table S4. The relationships between chemotherapy regimens and responses in 63 NSCLC patients

	N	Chemotherapy response		*P*
		**Chemo-sensitive**	**Chemo-resistant**	
**Total cases**	63	32	31	
**Containing pemetrexed (with platinum)**	36	21	15	0.1669
**Without pemetrexed (platinum plus paclitaxel [14], plus gemcitabine [8], plus vinorelbine [5])**	27	11	16	

N, number. Analyses were performed using the χ^2 test, $^*p < 0.05$.

Table S5. Multidrug sensitivities of the two PEM-R NSCLC cell lines and their parental cell lines

Drug	IC50		Resistance index	*p*	IC50		Resistance index	*p*
	**H1299**	**H1299/PEM**			**A549**	**A549/PEM**		
Pemetrexed	0.66±0.13 (μM)	14.33±1.74 (μM)	23.99±3.80	0.0079**	1.15±0.23 (μM)	25.28±4.42 (μM)	23.51±2.90	0.0079**
Taxel	1.92±0.37 (nM)	18.24±4.60 (nM)	9.66±1.59	0.0079**	3.18±0.35 (nM)	4.77±0.46 (nM)	1.62±0.30	0.0556
Gemcitabine	0.13±0.03 (μM)	0.33±0.09 (μM)	3.26±1.02	0.0952	2.54±0.80 (μM)	4.67±0.75 (μM)	2.42±0.75	0.0556
5-fluorouracil	3.72±1.04 (μM)	46.30±5.68 (μM)	15.43±3.20	0.0079**	2.00±0.09 (μM)	40.59±2.52 (μM)	20.16±1.68	0.0079**
Docetaxel	1.84±0.77 (nM)	2.49±0.85 (nM)	2.08±0.74	0.3095	1.93±0.41 (nM)	9.29±1.35 (nM)	4.12±1.17	0.0317*
Carboplatin	8.10±0.96 (μM)	65.12±4.81 (μM)	8.62±1.39	0.0079**	12.20±0.94 (μM)	135.78±6.49 (μM)	11.47±1.25	0.0079**
Cisplatin	1.01±0.19 (μM)	13.86±2.64 (μM)	14.23±2.16	0.0079**	0.79±0.13 (μM)	10.07±2.17 (μM)	13.28±2.30	0.0079**

IC50: 50% inhibitory concentration. Sensitivities of the NSCLC cells to the drugs were determined using the CCK-8 assay. The resistance index represents the ratio of the IC50 in the PEM-R cell to the IC50 in the parental cell for each drug. Statistical analyses were performed using the Mann-Whitney test (n = 5), $^{*}p < 0.05$ or $^{**}p < 0.01$.

Supplement figure 1

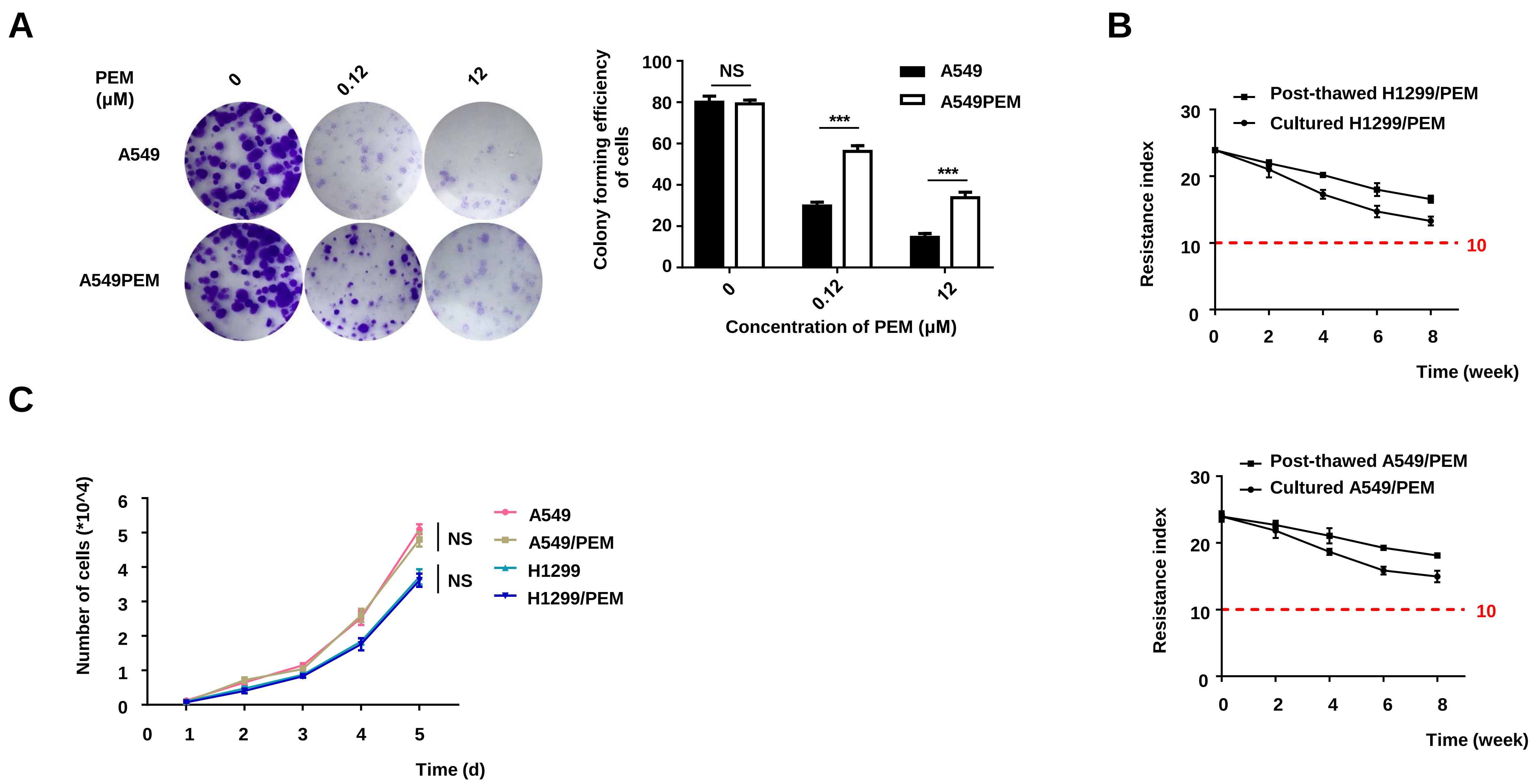

Supplement figure 2

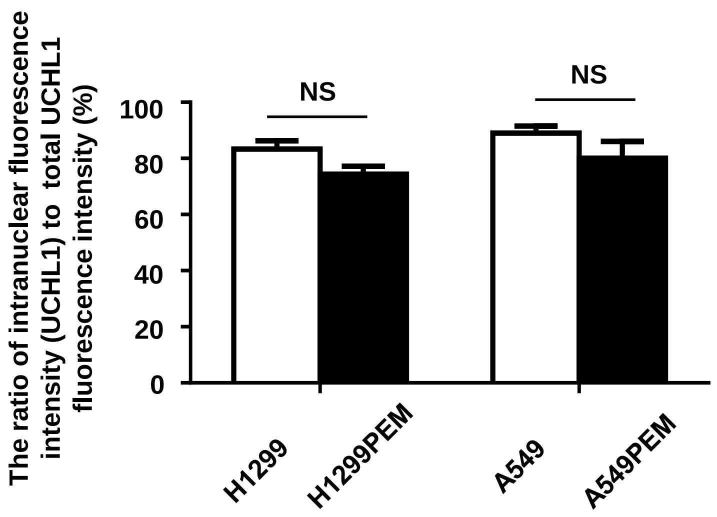

Supplement figure 3

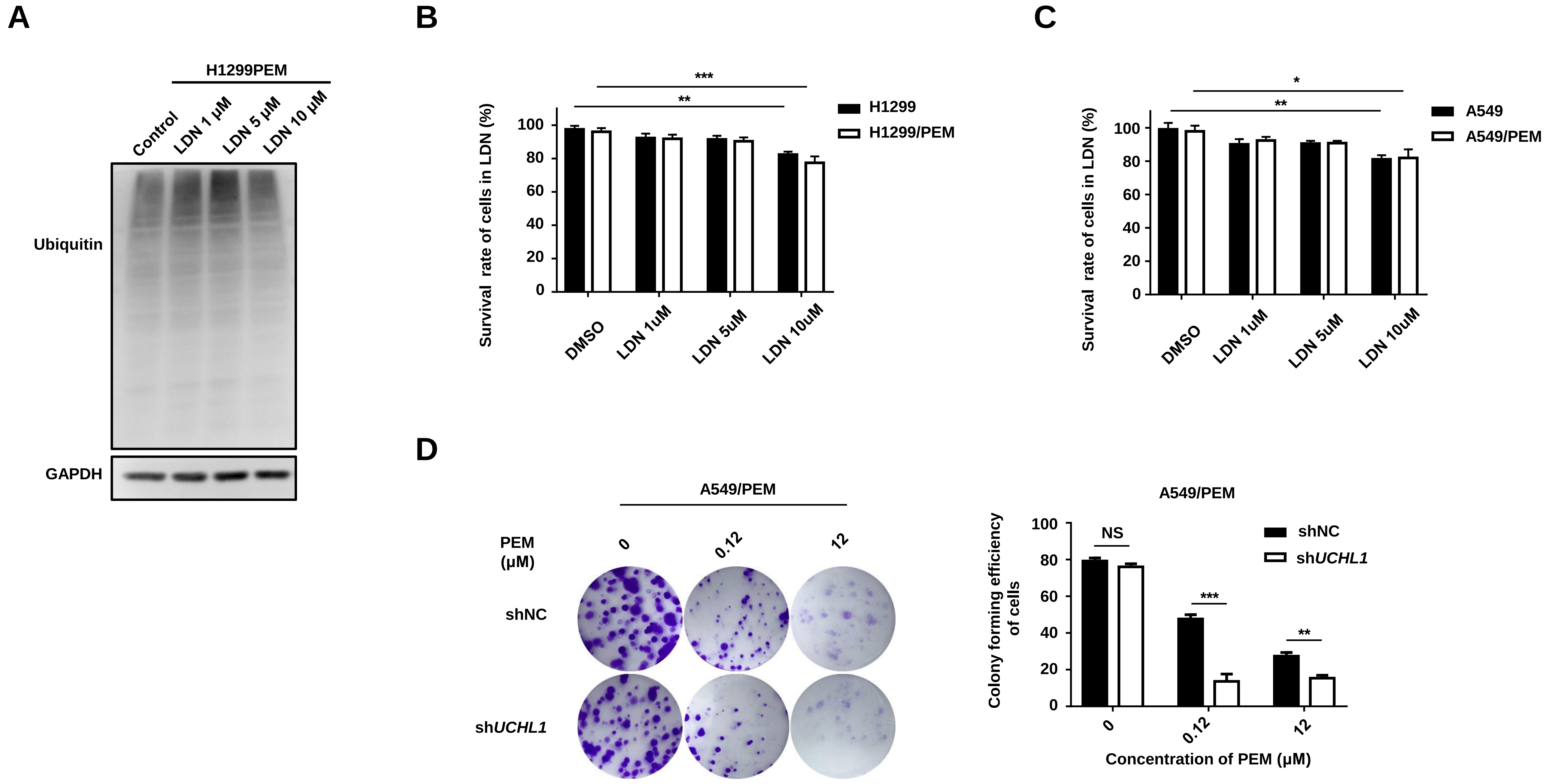

Supplement figure 4

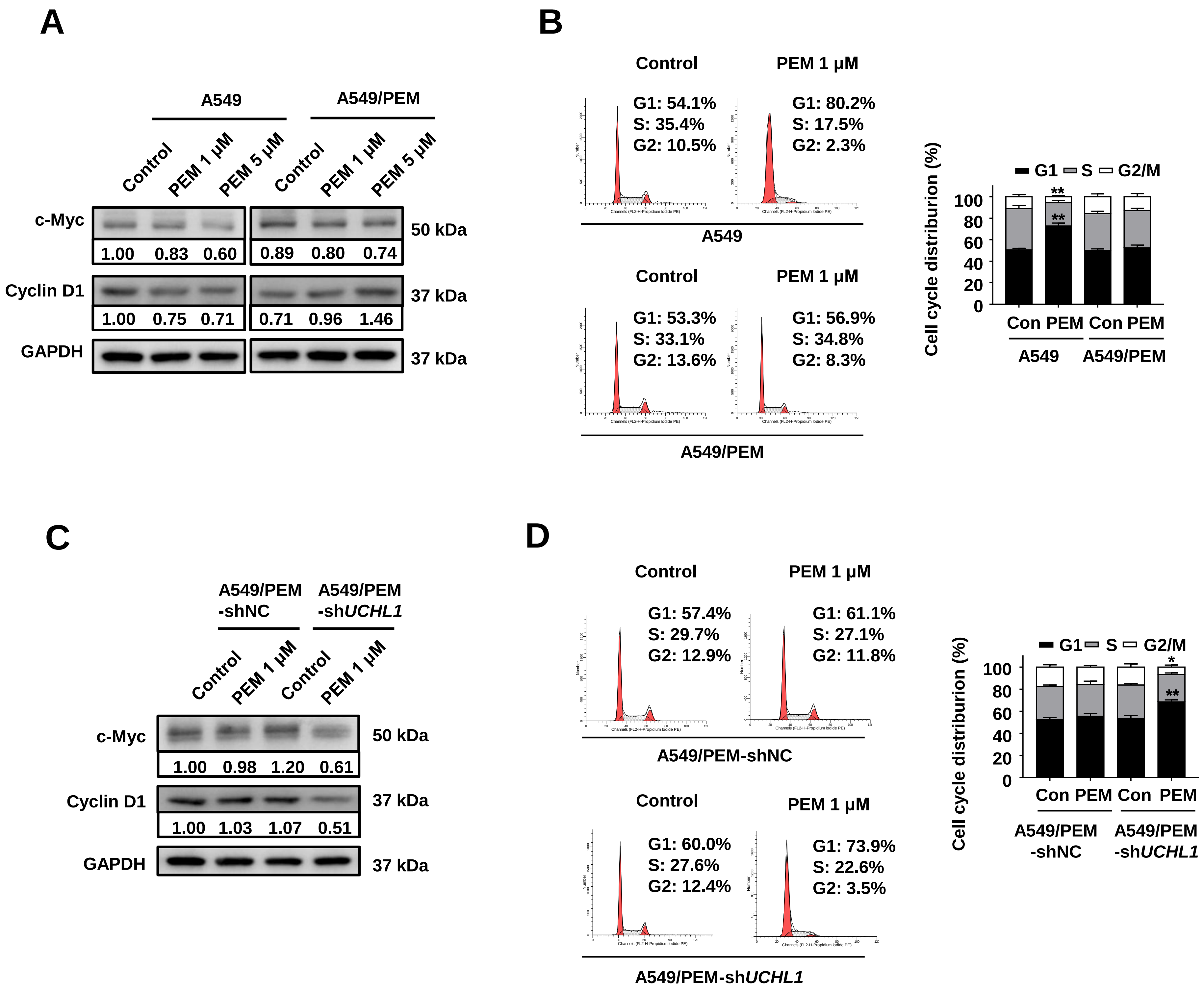

Supplement figure 5

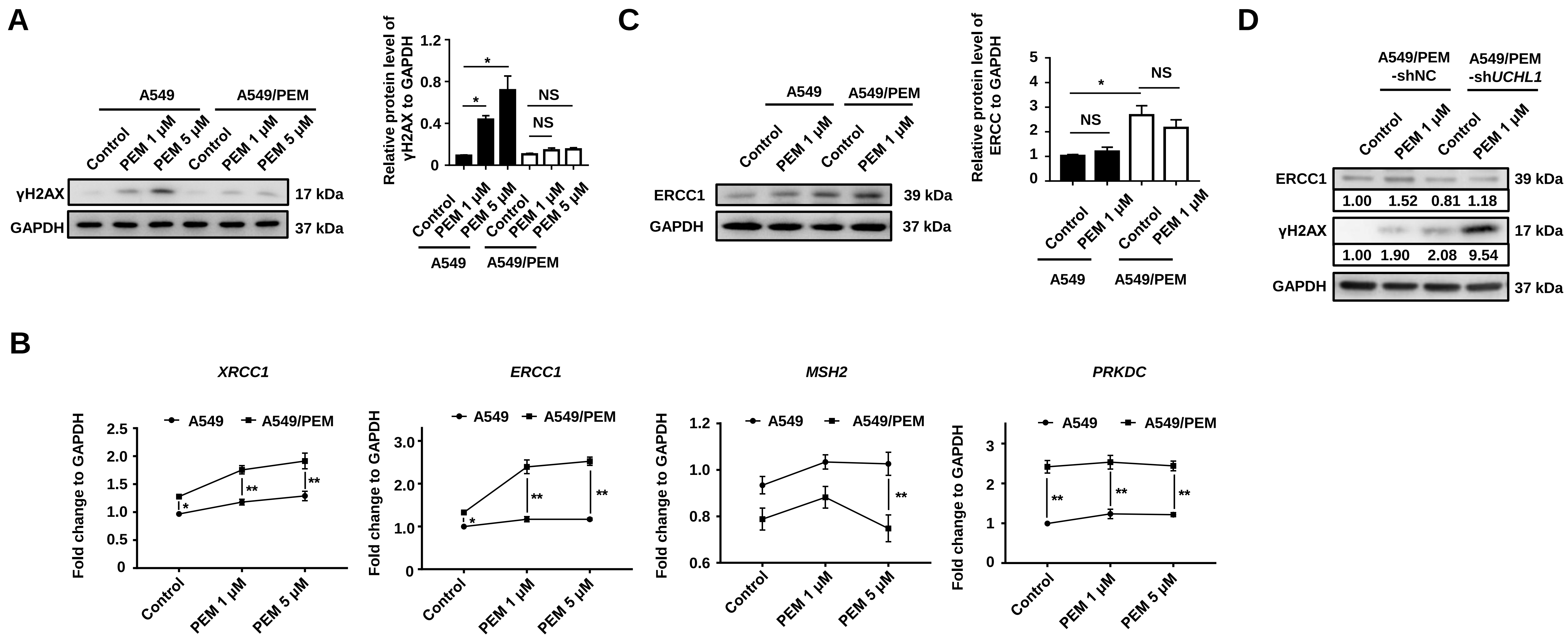

Supplement figure 6

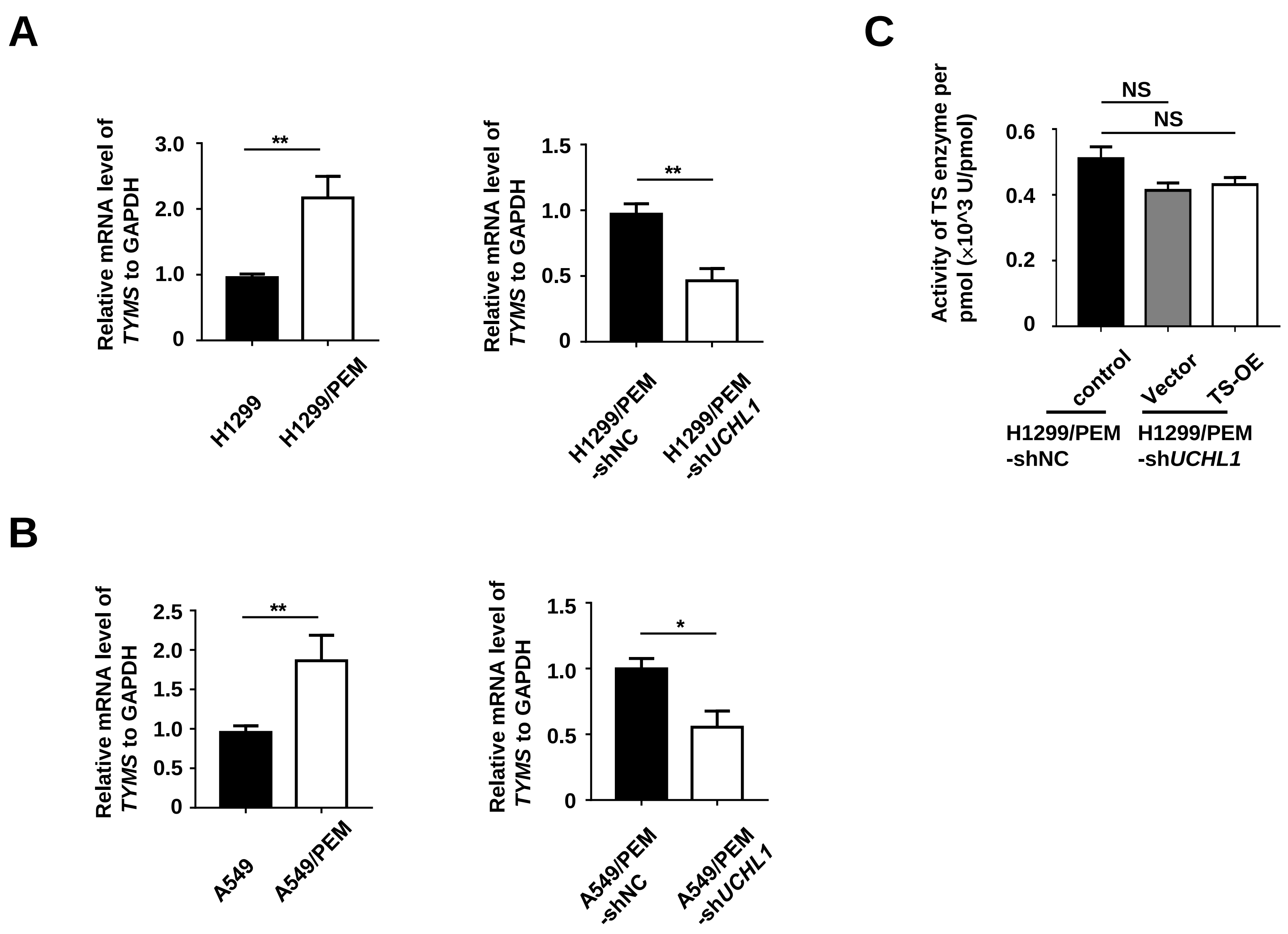

Supplement figure 7

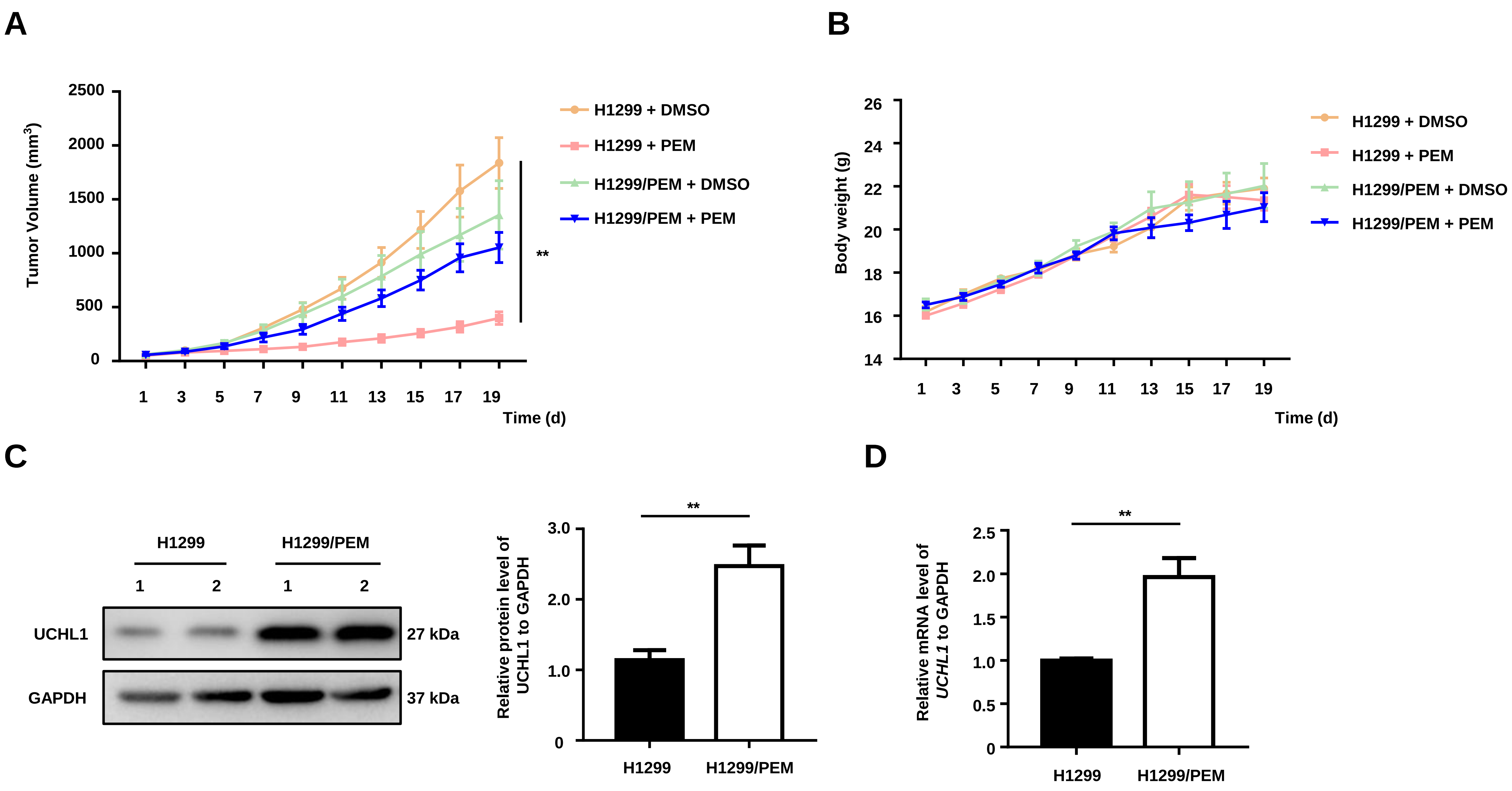

Supplement figure 8

A

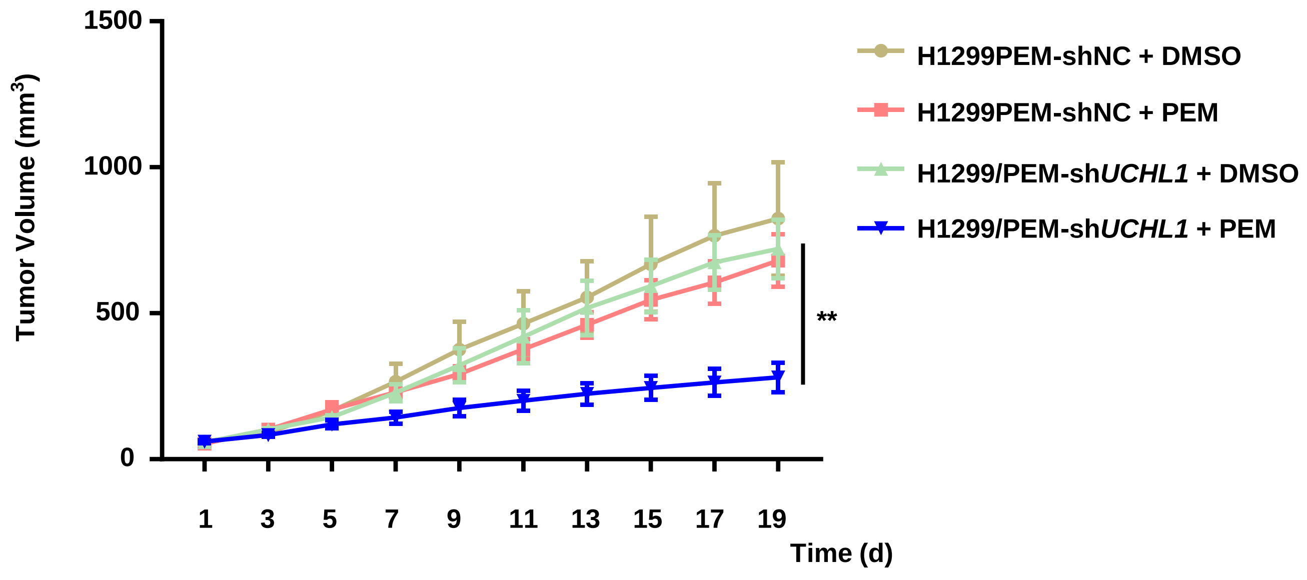

B

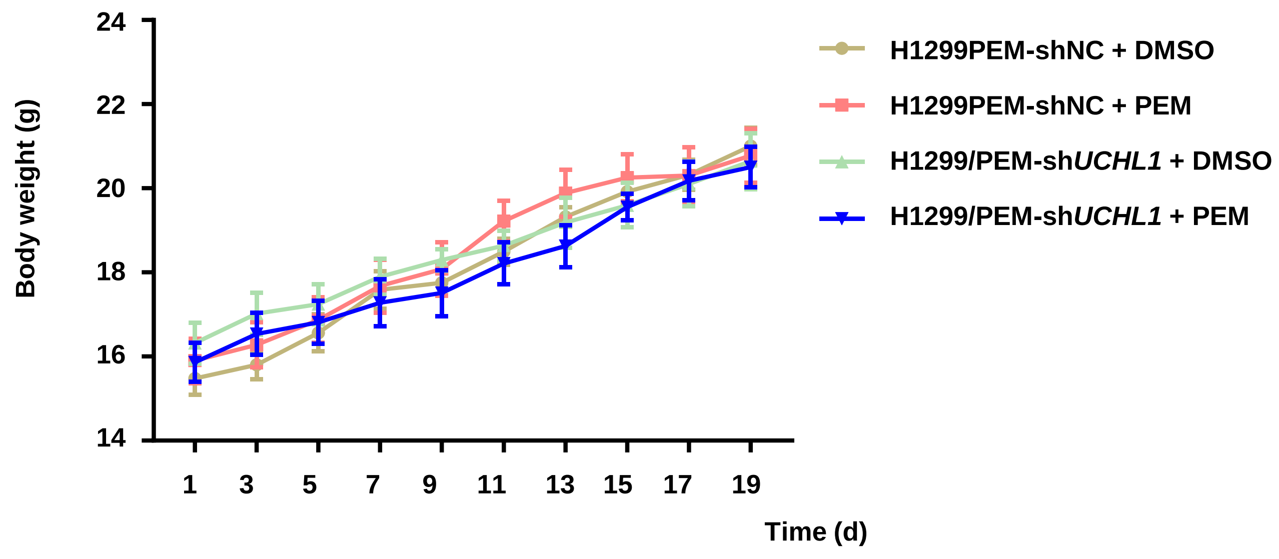

C

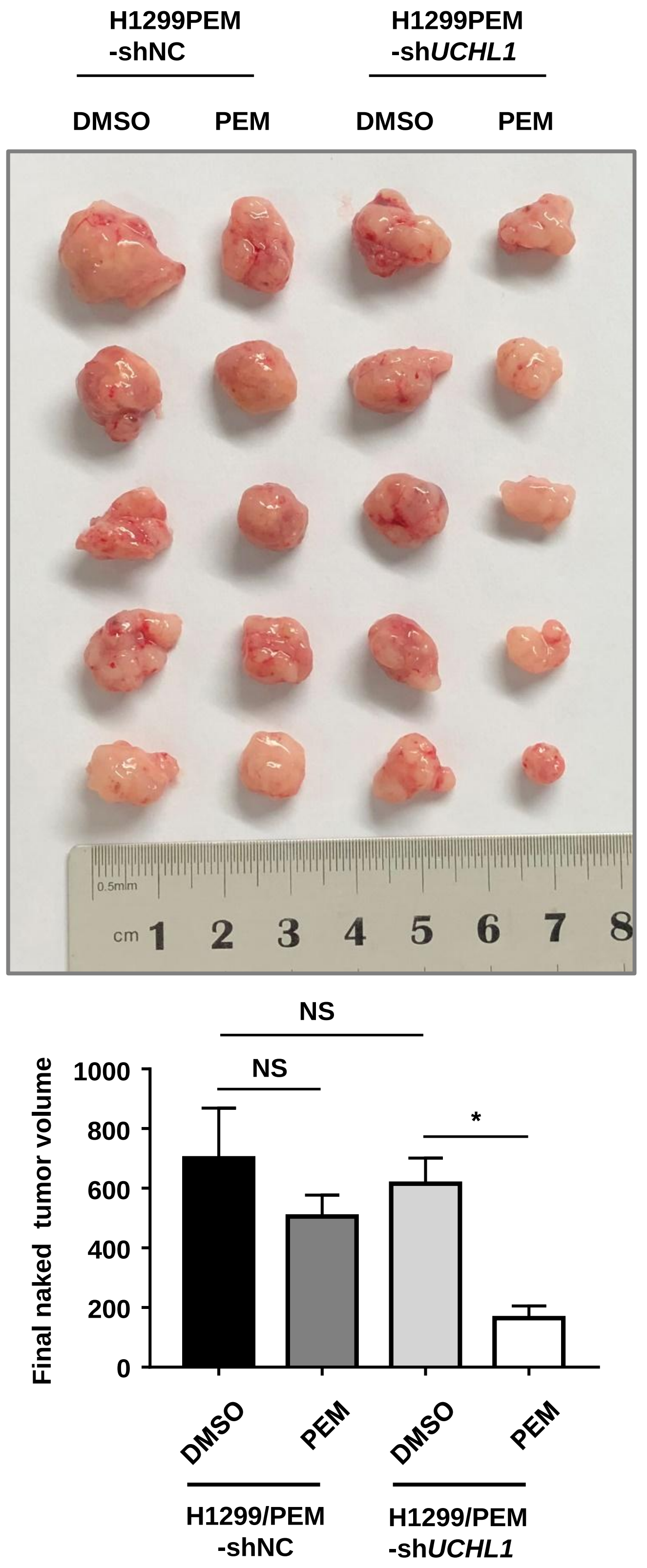

Supplement: Supplementary file 1 — Supplementary figures and tables. [file thnov10p6048s1.pdf]
